# Development of Therapeutic and Prophylactic Zinc Compositions for Use against COVID-19: A Glimpse of the Trends, Inventions, and Patents

**DOI:** 10.3390/nu14061227

**Published:** 2022-03-14

**Authors:** Mohd Imran, Waseem Fatima, A. Khuzaim Alzahrani, Nida Suhail, Mohammed Kanan Alshammari, Abdulrahman A. Alghitran, Fayez Nafea Alshammari, Mohammed M. Ghoneim, Sultan Alshehri, Faiyaz Shakeel

**Affiliations:** 1 Department of Pharmaceutical Chemistry, Faculty of Pharmacy, Northern Border University, Rafha 91911, Saudi Arabia; 2 Department of Clinical Nutrition, Faculty of Applied Medical Sciences, Northern Border University, Arar 91431, Saudi Arabia; fatimawaseem1512@gmail.com; 3 Department of Medical Laboratory Technology, Faculty of Applied Medical Sciences, Northern Border University, Arar 91431, Saudi Arabia; akaalz@nbu.edu.sa (A.K.A.); nsuhail123@gmail.com (N.S.); 4 Department of Pharmaceutical Care, Rafha Central Hospital, North Zone, Rafha 91911, Saudi Arabia; ii_kanan101@outlook.com; 5 Department of Clinical Pharmacy, General Administration of Pharmaceutical Care, Ministry of Health, Riyadh 11176, Saudi Arabia; alghitran.a@gmail.com; 6 Community Pharmacist, Al-Dawaa Pharmacies, Kingdom of Saudi Arabia, Hafer Albatin 39911, Saudi Arabia; fayezalshammari96@gmail.com; 7 Department of Pharmacy Practice, College of Pharmacy, AlMaarefa University, Ad Diriyah 13713, Saudi Arabia; mghoneim@mcst.edu.sa; 8 Department of Pharmaceutics, College of Pharmacy, Riyadh 11451, Saudi Arabia; salshehri1@ksu.edu.sa

**Keywords:** zinc, SARS-CoV-2, dosage forms, prophylaxis, treatment, clinical trial

## Abstract

Zinc is an essential nutrient for human health; it is involved in the catalytic, structural, and regulatory functions of the human cellular system. Different compositions of zinc, as well as its pharmaceutically acceptable salts, are available on the market. Recent studies have demonstrated the role of zinc in combating COVID-19. It has been determined that zinc prevents the entry of SARS-CoV-2 into cells by lowering the expression of ACE-2 receptors and inhibiting the RNA-dependent RNA polymerase of SARS-CoV-2. Zinc also prevents the cytokine storm that takes place after the entry of SARS-CoV-2 into the cell, via its anti-inflammatory activity. The authors believe that no study has yet been published that has reviewed the trends, inventions, and patent literature of zinc compositions to treat/prevent COVID-19. Accordingly, this review has been written in order to fill this gap in the literature. The information about the clinical studies and the published patents/patent applications was retrieved from different databases. This review covers patent literature on zinc compositions up to 31 January 2022. Many important patents/patent applications for zinc-based compositions filed by innovative universities and industries were identified. The patent literature revealed zinc compositions in combination with zinc ionophores, antioxidants, antivirals, antibiotics, hydroxychloroquine, heparin, ivermectin, and copper. Most of these studies were supported by clinical trials. The patent literature supports the potential of zinc and its pharmaceutical compositions as possible treatments for COVID-19. The authors believe that countless zinc-based compositions are still unexplored, and there is an immense opportunity to evaluate a considerable number of the zinc-based compositions for use against COVID-19.

## 1. Introduction

Severe acute respiratory syndrome coronavirus 2 (SARS-CoV-2) is the causative virus for the contagious coronavirus disease of 2019 (COVID-19). The first SARS-CoV-2 infection case was identified in December 2019 in Wuhan, China [1]. The symptoms of COVID-19 (i.e., dry cough, fever, fatigue, headache, nasal congestion, sore throat, muscle/joint pain, breathlessness, loss of taste/smell, diarrhea, etc.) may start 1–14 days after SARS-CoV-2 infection [2]. The majority of COVID-19 patients (~80%) recover without needing hospital treatment, ~15% get seriously ill, and ~5% need intensive care. The COVID-19 complications causing death comprise acute respiratory distress syndrome (ARDS), respiratory failure, thromboembolism, septic shock, and/or multiorgan failure [3].

As of 31 January 2022, the World Health Organization (WHO) reported 373,229,380 confirmed COVID-19 cases, 5,658,702 COVID-19-related deaths, and 9,901,135,520 vaccine doses taken [4]. The COVID-19 pandemic has caused an expressive impact on the quality of life, the economy, and considerable serious complications among the immunocompromised population worldwide [5]. SARS-CoV-2 infection can be contained by medicines, vaccines, and a healthy immune system. Some medicines for COVID-19 have been developed, such as remdesivir [6], Paxlovid [7], and molnupiravir [8]. Scientists have also developed vaccines (e.g., the Moderna COVID-19 Vaccine, Pfizer BioNTech COVID-19 Vaccine, and Comirnaty) against COVID-19 [9]. The immune system of a person can be stimulated/boosted through the appropriate use of minerals (zinc and copper), vitamins (vitamin C and vitamin D), and a diet rich in immunostimulant nutrients. Zinc is an established essential trace element and nutrient for human health [10].

Recently, the role of zinc in the prevention and treatment of COVID-19 has been established and documented in published reviews [11,12,13,14,15,16,17,18,19,20]. However, the previous reviews are silent about the trends, inventions, and patent literature of innovative universities and pharmaceutical companies that are working on the development of zinc-based compositions to combat COVID-19. This review has been written to fill this gap, and will be useful to the scientific community working on the development of zinc-based compositions for use against COVID-19.

## 2. Zinc

### 2.1. Importance of Zinc

Zinc, a brittle and silver/grey metal in its unoxidized form, is represented by the symbol Zn (atomic number 30); it is the second most abundant metal in the human body, after iron [21]. Zinc is an essential nutrient because it is needed in almost all aspects of cellular/biological processes (i.e., catalytic functions, structural functions, and regulatory functions) of the human body [22]. The catalytic functions of zinc are attributed to its antioxidative nature, and to it being an essential component of ~3000 catalytic enzymes (e.g., dismutase, dehydrogenase, anhydrase, carboxypeptidase, aminopeptidase, lyase, metalloproteinase, S-methyltransferase, carboxytransferase, deacylase, hydrolase, nuclease, synthase, etc.) [22,23]. The structural functions of zinc include maintenance and stabilization of the intracellular components/cell membranes [22,23]. The regulatory functions of zinc encompass being a component of DNA/RNA polymerase, many kinases, and ribonuclease [22,23].

**Figure 1:**
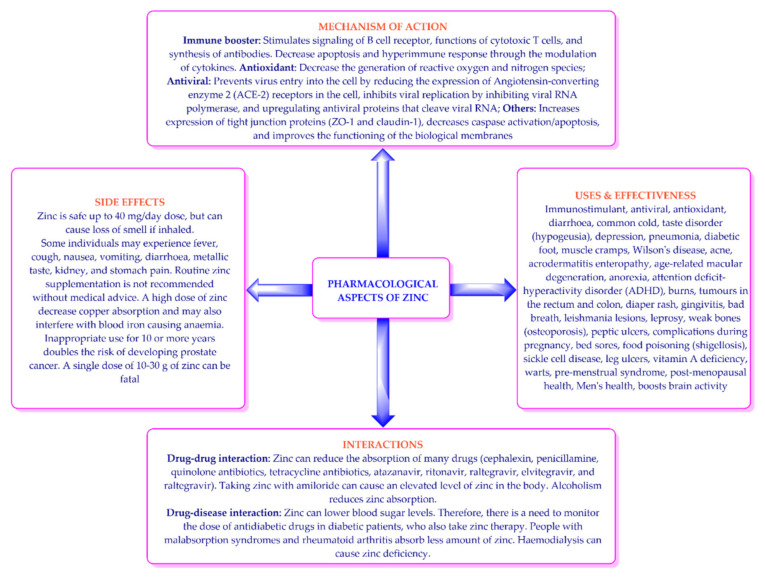
Mechanisms of action, uses, side effects, and interactions of zinc.

The human body does not have a zinc store, although it can reutilize some of the zinc released after tissue catabolism [24]. Accordingly, zinc should be consumed regularly in the diet—especially through foods of animal origin (e.g., meat, shellfish, etc.)—or as a supplement/medicine in order to prevent its deficiency [22,23,24]. The absorption of zinc increases in a diet containing protein, histidine, methionine, and citrate. However, some drugs (Figure 1) and compounds/elements (e.g., phytic acid, cadmium, high doses of iron, and casein) lower the bioavailability of zinc by complexing with it [24,25,26,27]. Accordingly, it is imperative to identify compounds that increase or decrease the absorption of zinc during zinc therapy. The daily zinc requirements for different populations are listed in Table 1 [26,27].

After ingestion, zinc is released as Zn^+2^ ions and absorbed up to an extent of 33% in a normal healthy person, wherein a decrease in zinc absorption takes place with an increase in age. The zinc is transported to cells through transport proteins [24,25], where it demonstrates its biological effects, including antioxidant, immunostimulant, and antiviral effects (Figure 1). Zinc deficiency can cause many effects, including growth hindrance, delayed sexual development, skin problems, diarrhea, alopecia, decreased appetite, osteoporosis, and increased susceptibility to viral infections. Accordingly, zinc supplements and zinc-based medicines are effective in the treatment of many illnesses (Figure 1). At the same time, excessive use of zinc can cause many side effects, and can demonstrate drug–drug and drug–disease interactions (Figure 1) [24,25,26,27].

### 2.2. USFDA-Approved Prescription Zinc Compositions

A search for United States Food and Drug Administration (USFDA)-approved zinc products was conducted on the Orange Book of the USFDA [28] on 1 February 2022, using “zinc” as the keyword. A total of 40 products were obtained, out of which 21 were prescription products and 19 have been discontinued. Among the 21 products, 10 belonged to bacitracin zinc salts, and 11 belonged to zinc sulfate (3 products), zinc chloride (2 products), zinc acetate (2 products), and zinc combinations with other salts/drugs (4 products). The results of the search are provided in Table 2. The USFDA-approved zinc products mentioned in Table 2 are indicated for the parenteral/oral intake of zinc, for maintaining zinc serum levels and avoiding any zinc deficiency syndrome, and for diaper dermatitis (ointment).

### 2.3. Zinc and SARS-CoV-2

**Figure 2:**
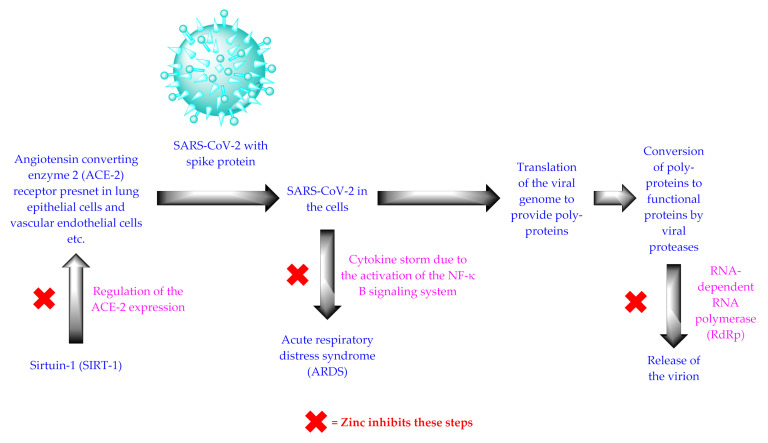
The life cycle of SARS-CoV-2.

SARS-CoV-2 is an RNA virus containing a spike protein. The virus enters human cells after binding its spike protein with the ACE-2 receptors, which are expressed in many human cells (e.g., lung epithelial cells, vascular endothelial cells, etc.) [6,8]. Zinc minimizes the activity of sirtuin-1 (SIRT-1), which regulates ACE-2 expression. Accordingly, zinc prevents the entry of SARS-CoV-2 into the cell by lowering the expression of the ACE-2 receptors [29] (Figure 2). SARS-CoV-2 also needs RNA-dependent RNA polymerase (RdRp) for its replication. Zinc inhibits the RdRp and prevents the replication of SARS-CoV-2 (Figure 2) [6,8]. The principal hallmark of COVID-19 infection is an imbalance of the immune system. The intracellular presence of SARS-CoV-2 generates a cytokine storm due to the activation of the NF-κB signaling system, and causes ARDS [30,31]. Zinc demonstrates its anti-inflammatory effect by suppressing the NF-κB signaling and attenuating the cytokine storm [30,31] (Figure 2). Zinc also stimulates the generation of interferons that help in the synthesis of antiviral proteins (i.e., latent ribonuclease and protein kinase) that degrade the RNA of SARS-CoV-2 [22]. Zinc increases the activity of natural killer cells, cytotoxic cells, and B-cell receptor signaling, and increases the production of antibodies. All of these factors make zinc an excellent remedy against COVID-19 [32].

### 2.4. Clinical Trials of Zinc Compositions for the Treatment/Prevention of COVID-19

A search on the clinical trials database [33] was conducted on 31 January 2022 using the keyword “zinc” in combination with other keywords. This search provided many studies on zinc compositions as treatments for COVID-19 (zinc = 2086 studies; COVID-19 = 7337 studies; zinc + COVID-19 = 68 studies; zinc + COVID = 68 studies; zinc + SARS-CoV-2 = 19 studies; zinc + coronavirus disease 2019 = 4 studies; zinc + coronavirus disease 19 = 2 studies; zinc + severe acute respiratory syndrome coronavirus 2 = 2 studies; zinc + 2019-nCoV = 0 studies; zinc + novel coronavirus = 0 studies; zinc + SARS coronavirus 2 = 0 studies; zinc + Wuhan coronavirus = 0 studies). The interventional clinical studies (Phases 1, 2, 3, and 4) based on zinc compositions are summarized in Table 1. The complete details of the clinical trials mentioned in Table 3 can be obtained using the NCT numbers in the clinical trials database (https://www.clinicaltrials.gov/ accessed on 31 January 2022).

## 3. Patent Searching

The patent exploration was carried out utilizing different patent databases (Sci-Finder, Espacenet, Patentscope, and the USPTO) [34,35,36,37,38,39,40] on 31 January 2022. A combination search for the terms “Zinc + COVID-19” and “Zinc + SARS-CoV-2” was performed in different fields (claims, abstract, and title) of Espacenet, Patentscope, and the USPTO. A similar search was performed in the “Research topic” field of Sci-Finder. The Espacenet database (zinc + COVID-19 = 111 hits; zinc + SARS-CoV-2 = 110 hits), Patentscope database (zinc + COVID-19 = 117 hits; zinc + SARS-CoV-2 = 127 hits), USPTO database (zinc + COVID-19 = 39 hits; zinc + SARS-CoV-2 = 27 hits), and Sci-Finder (zinc + COVID-19 = 155 hits; zinc + SARS-CoV-2 = 65 hits) resulted in a total of more than 150 hits. The duplicate patent references were removed and segregated according to their families.

## 4. Patent Analysis

A patent has two types of claims: independent claims, and dependent claims. The components of the independent claims are the essential parts of the invention. Accordingly, all of the patents/patent applications whose independent claims relating to the use of zinc compositions to treat/prevent COVID-19/SARS-CoV-2 infection were included in this review, and are summarized in Table 4. The patents/patent applications for zinc-based masks, polymers, fabrics, coatings, and devices were excluded from this review.

Our search also revealed some patents/patent applications that implicitly relate to the use of zinc-based compositions to treat/prevent COVID-19/SARS-CoV-2 infection. However, these documents are silent about the anti-COVID-19/antiviral activity of zinc. These documents include US20210244726A1 (a composition of chloroquine/hydroxychloroquine, a macrolide antibiotic, and zinc) [70], IN202021031040A (an Ayurvedic composition containing zinc) [71], CN111450100A (a composition of caffeine, chlorogenic acid, and zinc) [72], WO2021074706A1 (an immunomodulatory composition of many nutrients and zinc) [73], WO2021183456A1 (a composition of lenzilumab and zinc) [74], WO2021186396A2 (a composition of artemisinin and zinc) [75], WO2021195017A1 (an iodine inhalation composition containing zinc) [76], US20210346453A1 (a composition of *Uncaria tomentosa* extract containing zinc) [77], WO2021224356A1 (a composition of plerixafor/burixafor containing zinc) [78], US20210338765A1 (a composition of ashwagandha, shallaki, ginger, turmeric, and zinc) [79], US20210330587A1 (an oral sanitizer containing zinc) [80], WO2021205242A1 (a composition of a synbiotic and zinc) [81], WO2021205437A1 (a composition of a cholesterol-lowering compound, an S-adenosylhomocysteine hydrolase inhibitor, a DOTH inhibitor, and zinc) [82], and FR3109299A1 (a composition of artemether, azithromycin, and zinc) [83].

## 5. Conclusions

Zinc possesses an appreciable antiviral activity, and its effects against SARS-CoV-2 are also well documented. The clinical data of zinc and its compositions show promise against COVID-19. The patent literature also supports the potential of zinc and its pharmaceutical compositions as possible treatments for COVID-19. The identification of the inventive composition of zinc indicates many foreseeable commercially available zinc-based medicines to combat COVID-19. However, the authors trust that many zinc-based compositions are still unexplored, and there is great scope to assess a large number of the zinc-based compositions for use against COVID-19.

## 6. Discussion

Zinc, an essential nutrient for human health, has been used in clinical practice as a medicine, immune booster, and food supplement (Figure 1) [10,14]. SARS-CoV-2 (RNA virus) enters human cells via the ACE-2 receptors, and requires RdRp for its replication [6,8]. RdRp is a major target for antiviral drug research. Studies have shown that increased intracellular concentration of zinc interferes with the proteolytic processing of polyproteins in several RNA viruses. Increased intracellular concentrations of zinc have also demonstrated inhibition of the isolated RdRp complex, purified recombinant RdRp, and the replication of coronavirus in tissue culture [41]. The zinc ions also suppress/lower the expression of the ACE-2 receptors, preventing the entry of SARS-CoV-2 into the cells [29]. Therefore, zinc demonstrates the dual character of prophylactic and therapeutic intervention for COVID-19.

Zinc and its compositions are in clinical trials to prevent and treat COVID-19 (Table 3). The data shown in Table 3 also reveal 3 studies in phase 1, 9 studies in phase 2, 12 studies in phase 3, and 4 studies in phase 4. Among these 28 studies, 18 studies are related to the treatment of COVID-19, 5 are related to the prevention of COVID-19, 4 are related to the use of zinc for supportive care, and 1 study relates to post-COVID-19 smell and taste dysfunction. Seven studies were conducted in the United States; one each in Iraq, Indonesia, Singapore, Thailand, Canada, Australia, and Pakistan; two in India; four each in Saudi Arabia and Egypt; three in Tunisia, and one study’s location is undisclosed. Most of the clinical studies [26] have been planned as randomized clinical trials. It is also important to note that only one study (NCT04475588) has revealed its clinical outcomes. Two studies (NCT04590274 and NCT04528927) have been withdrawn. The literature also includes a clinical study (NCT04342728) related to the use of zinc gluconate, vitamin C, and their combination to shorten the treatment of COVID-19 [84]. However, the results of this study were not encouraging; therefore, it has been stopped for ineffectiveness. The compositions of zinc mentioned in Table 3 consist of one or more drugs/elements selected from hydroxychloroquine, azithromycin, vitamin C, vitamin D, N-acetylcysteine, elderberry, quercetin, ivermectin, doxycycline, insulin, gabapentin, itolizumab, heparin, acetaminophen, dexamethasone, chlorine dioxide, famotidine, lactoferrin, green tea extract, vitamin B12, methylene blue solution, phenformin, resveratrol, selenium, extract of *Psidii guava*, nitazoxanide, ribavirin, chloroquine, darunavir, ritonavir, and bromelain. The results of one clinical study (NCT04322513) have demonstrated the role of vitamin D against COVID-19 [85]. Accordingly, the important combination of zinc and vitamin D may provide synergistic effects to deal with COVID-19.

**Figure 3:**
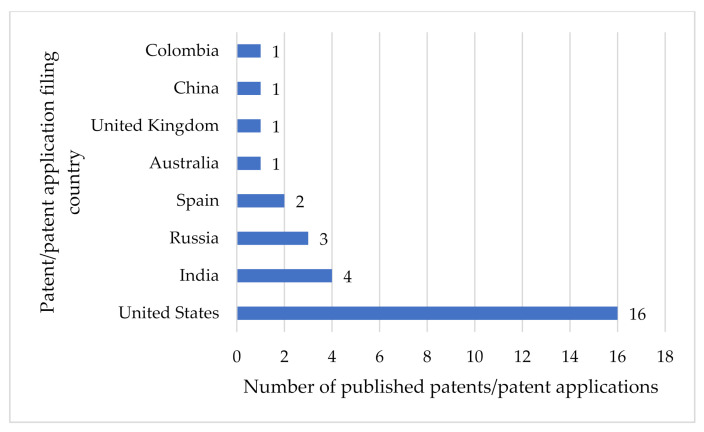
Patent-filing countries.

Our search revealed many patents/patent applications for pharmaceutical compositions of zinc for use against COVID-19 (Table 4). Table 4 provides a summary of 29 patents/patent applications, of which 2 were published in 2020, 26 in 2021, and 1 in 2022. This pattern is expected, because the first COVID-19 case was identified in December 2019, and scientists started working on COVID-19 treatments in 2020. The majority of these patent applications have been filed in the United States (16), followed by India (4), Russia (3), Spain (2), Australia (1), the United Kingdom (1), China (1), and Colombia (1) (Figure 3).

It is interesting to note that most of these compositions or their equivalent compositions are in clinical trials (Table 3). In almost all of the clinical studies and patent specifications, zinc has been used in the form of its salts (zinc acetate, zinc sulfate, zinc lactate, zinc aspartate, zinc undecylenate, zinc pyrithione, zinc gluconate, zinc oxide, zinc picolinate, zinc iodide, zinc chloride, zinc citrate, zinc carbonate, zinc hydroxide, zinc fluoride, zinc bromide, zinc sulfonate, zinc glucuronate, zinc bisglycinate, and zinc pyruvate). Remdesivir [6], Paxlovid [7], and molnupiravir [8] are approved treatments for COVID-19. However, no clinical study or patent literature has been found relating to the specific combination of zinc with remdesivir, Paxlovid, or molnupiravir. This can be considered as an opportunity for further research of treatments for COVID-19. However, the possibility of the interaction of zinc with antiviral drugs must be assessed before COVID-19 treatment, as zinc has been reported to interfere with the absorption of many antivirals (Figure 1). A zinc ionophore helps in the transport of zinc at the target site, such as a cell. Many clinical studies and patent applications have used compositions of zinc with a zinc ionophore (e.g., alpha defensin 5, quercetin, curcumin and epigallocatechin gallate, silymarin, hesperidin, diosmin, neral, geranial, and citronellal). High doses of zinc or its regular intake may cause copper deficiency or side effects [52]. The targeted delivery of zinc utilizing a zinc ionophore may reduce these effects.

Hypertensive people are considered to be at risk of COVID-19. ACE-2, a metalloenzyme, is implicated in the pathogenesis of hypertension. Zinc lowers the expression of ACE-2, and is also an immune booster; therefore, it is believed that zinc compositions may provide better therapeutic outcomes when used along with antihypertensive ACE inhibitors (e.g., captopril, lisinopril, ramipril, etc.) [12]. The use of zinc compositions for enriching the human immune system and to prevent the secondary complications of COVID-19 in high-risk populations (e.g., geriatric/immunocompromised patients, patients suffering from chronic diseases, medical and paramedical professionals, etc.) has also been claimed [56].

Despite the many benefits associated with zinc, some studies have warned against the excessive use of zinc among COVID-19 patients [86,87]. Zinc is needed for the growth of many pathological fungi, including Mucorales, which cause fatal mucormycosis [86,88]. Some others have reported that zinc might have a negative impact, and high zinc levels in hepatitis can increase the viral load by blocking the production of interferons (IFN-λ) [89], induce apoptosis in T cells, B cells, and thymocytes [90,91], and lead to copper deficiency, which can cause anemia and leukopenia [92].

Zinc can provide positive or negative effects based on the condition of the consumer/patient. However, zinc supplements are not expensive, and are readily available on the market. This facilitates the overuse of zinc supplements among COVID-19 patients. Accordingly, zinc supplements must be used under medical supervision. Most of the literature supports the notion that zinc can provide beneficial effects among mild-to-moderate COVID-19 patients, wherein the dose, duration of zinc treatment, and condition of the patient are important aspects during COVID-19 therapy. Accordingly, many inventions of zinc-based compositions have been developed for use against COVID-19. The identification of the inventive composition and current clinical trials of zinc-based compositions indicates many foreseeable commercially available zinc compositions to combat COVID-19. However, there is a need to assess the efficacy of zinc supplements and inventive compositions among severe COVID-19 cases requiring intensive care [87].

## Figures and Tables

**Table 1:** Average requirements of zinc.

Age Groups	FNB *		IZNCG **	
	Reference Weight (kg)	Requirement (mg/Day)	Reference Weight (kg)	Requirement (mg/Day)
6–12 months	9	0.84	9	0.84
1–3 years	13	0.74	12	0.53
4–8 years	22	1.20	21	0.83
8–13 years	40	2.12	38	1.53
14–18 years (Male)	64	3.37	64	2.52
14–18 years (Female)	57	3.02	56	1.98
Pregnancy	-	4.1–5.0	-	2.68
Lactation	-	3.8–4.5	-	2.98

* Food and Nutrition Board; ** International Zinc Nutrition Consultative Group.

**Table 2:** USFDA-approved zinc prescription products.

Active Ingredient (Proprietary Name; Applicant)	Application Number (Dosage Form; Route)	Strength
Zinc sulfate (Zinc sulfate; American Regent, Inc., New York, NY, USA)	N209377 (Solution; Intravenous)	10 mg base/10 mL
		25 mg base/5 mL
		30 mg base/10 mL
Zinc chloride (Zinc chloride in a plastic container; Hospira Inc., Illinois, IL, USA)	N018959 (Injectable; Injection)	1 mg zinc/mL
Zinc chloride (Zinc chloride; Exela Pharma Sciences, Lenoir City, Teen, USA)	A212007 (Injectable; Injection)	1 mg zinc/mL
Zinc acetate (Galzin; Teva Pharmaceuticals USA Inc., Fairfield, NJ, USA)	N020458 (Capsule; Oral)	25 and 50 mg base/capsule
Miconazole nitrate; Petrolatum white; Zinc oxide (Vusion; Mylan Pharmaceuticals Inc., Morgantown, WV, USA)	N021026 (Ointment; Topical)	0.25%; 81.35%; 15%
Cupric sulfate; Manganese sulfate; Selenious acid; Zinc sulfate (Tralement; American Regent Inc., New York, NY, USA)	N209376 (Solution; Intravenous)	3 mg base/mL and 3 mg base/5 mL along with other components
Cupric sulfate; Manganese sulfate; Selenious acid; Zinc sulfate (Multrys; American Regent Inc., New York, NY, USA)	N209376 (Solution; Intravenous)	1000 mcg/mL along with other components

**Table 3:** Interventional clinical studies on zinc compositions for the treatment of COVID-19.

Zinc Composition/Combination	Primary Purpose (Phase; Status; Results)	NCT Number (Allocation; Intervention Model; Completion Date)	Sponsor (Location of the Clinical Trial)
**Phase 1**			
Zinc + Hydroxychloroquine + Azithromycin + Vitamin C + Vitamin D3 + N-acetylcysteine + Elderberry + Quercetin	Prevention (1; Withdrawn; No results posted)	NCT04590274 (Not mentioned; Single Group Assignment; December 2021)	International Brain Research Foundation (Not mentioned)
Zinc sulfate + Ivermectin + Doxycycline + Vitamin D3 + Vitamin C	Treatment (1; Active, not recruiting; No results posted)	NCT04482686 (Randomized; Parallel Assignment; July 2022)	ProgenaBiome (United States)
Zinc + Insulin + Gabapentin + Ice cube stimulation	Post-COVID-19 smell and taste dysfunction (1; Not yet recruiting; No results posted)	NCT05104424 (Randomized; Parallel Assignment; 1 October 2022)	Ministry of Health (Saudi Arabia)
**Phase 2**			
Itolizumab + Supportive care (Zinc + Vitamins + Antivirals + Antibiotics + Hydroxychloroquine + Low-molecular-weight heparin + Steroids)	Treatment (2; Completed; Results posted)	NCT04475588 (Randomized; Parallel Assignment; 7 July 2020)	Biocon Limited (India)
Ivermectin + Doxycycline + Standard care (Zinc + Vitamin C + Acetaminophen + Vitamin D + Azithromycin + Dexamethasone)	Treatment (2; Completed; No results posted)	NCT04591600 (Randomized; Parallel Assignment; 14 October 2020)	Al-Karkh Health Directorate, Baghdad (Iraq)
Zinc acetate + chlorine dioxide + Famotidine + Lactoferrin + Green tea extract	Treatment (2; Recruiting; No results posted)	NCT04621149 (Randomized; Factorial Assignment; 31 March 2021)	Profact, Inc. (United States)
Zinc + Hydroxychloroquine + Azithromycin + Vitamin C + Vitamin D	Treatment (2; Recruiting; No results posted)	NCT04334512 (Randomized; Parallel Assignment; September 2024)	ProgenaBiome; DSCS CRO (United States)
Hydroxychloroquine + Vitamin C + Vitamin D + Zinc	Prevention (2; Recruiting; No results posted)	NCT04335084 (Randomized; Single Group Assignment; July 2025)	ProgenaBiome; DSCS CRO (United States)
Zinc citrate + Hydroxychloroquine + Azithromycin + Vitamin D + Vitamin B12	Treatment (2; Recruiting; No results posted)	NCT04395768 (Randomized; Parallel Assignment; 31 December 2021)	National Institute of Integrative Medicine (Australia)
Ivermectin + Zinc sulfate	Treatment (2; Recruiting; No result posted)	NCT04472585 (Randomized; Parallel Assignment; 30 October 2021)	Sheikh Zayed Federal Postgraduate Medical Institute (Pakistan)
Oral methylene blue solution + Inhaled phenformin + Zinc gluconate + Liquid potassium chloride in grape juice	Treatment (2; Not yet recruiting; No results posted)	NCT05003492 (Randomized; Parallel Assignment; November 2021)	Ministry of Health, Saudi Arabia; Kafr El Sheikh University, Egypt (Saudi Arabia)
Zinc picolinate + Resveratrol	Treatment (2; Active, not recruiting; No results posted)	NCT04542993 (Randomized; Single Group Assignment; June 2022)	Swedish Medical Center (United States)
**Phase 3**			
Zinc gluconate + Vitamin D	Supportive care (3; Recruiting; No results posted)	NCT04641195 (Randomized; Factorial Assignment; 31 March 2022)	Harvard School of Public Health; Foundation for Medical Research; University Health Network (India)
Ivermectin + Standard treatment (Azithromycin + Paracetamol + Vitamin C + Zinc + Lactoferrin + Anticoagulation)	Treatment (3; Not yet recruiting; No results posted)	NCT04937569 (Randomized; Sequential Assignment; 1 November 2021)	Assiut University (Egypt)
Zinc + Antioxidant supplements + Selenium	Supportive care (3; Recruiting; No results posted)	NCT04323228 (Randomized; Parallel Assignment; 30 December 2020)	King Saud University (Saudi Arabia)
Zinc + *Psidii guava* extract + Vitamin C	Treatment (3; Completed; No results posted)	NCT04810728 (Randomized; Parallel Assignment; 30 January 2021)	Faculty of Medicine Baiturrahmah University (Indonesia)
Zinc + Hydroxychloroquine + Azithromycin	Treatment (3; Withdrawn; No results posted)	NCT04528927 (Randomized; Parallel Assignment; 15 July 2020)	Abderrahmane Mami Hospital; Eshmoun Clinical Research Centre; Datametrix (Tunisia)
Zinc + Nitazoxanide + Ribavirin + Ivermectin	Treatment (3; Not yet recruiting; No results posted)	NCT04392427 (Randomized; Sequential Assignment;May 2022)	Mansoura University (Egypt)
Zinc + Vitamin C	Prevention (3; Completed; No results posted)	NCT04446104 (Randomized; Parallel Assignment; 31 August 2020)	National University Hospital (Singapore)
Zinc + Vitamin C (Dietary supplements)	Supportive care (3; Recruiting; No results posted)	NCT04780061 (Randomized; Parallel Assignment; March 2022)	The Canadian College of Naturopathic Medicine; Ottawa Hospital Research Institute; Vitazan Professional; New Roots Herbal (Canada)
Zinc + Doxycycline	Prevention (3; Completed; No results posted)	NCT04584567 (Randomized; Parallel Assignment; 1 November 2021)	Hedi Gharsallah; Dacima Consulting; General Administration of Military Health (Tunisia)
Zinc + Chloroquine	Treatment (3; Recruiting; No results posted)	NCT04447534 (Randomized; Parallel Assignment; 1 October 2030)	Tanta University (Egypt)
Ivermectin + Ribavirin + Nitazoxanide + Zinc	Treatment (3; Recruiting; No results posted)	NCT04959786 (Randomized; Parallel Assignment; December 2022)	Mansoura University (Egypt)
Zinc + Hydroxychloroquine	Prevention (3; Not yet recruiting; No results posted)	NCT04377646 (Randomized; Parallel Assignment; 31 July 2020)	Military Hospital of Tunis; Autoimmune Diseases Research Unit; Dacima Consulting (Tunisia)
**Phase 4**			
Ivermectin + Zinc sulfate vs. Hydroxychloroquine + Darunavir + Ritonavir + Zinc sulfate	Treatment (4; Recruiting; No results posted)	NCT04435587 (Randomized; Parallel Assignment; November 2021)	Mahidol University (Thailand)
Zinc sulfate	Treatment (4; Completed; No results posted)	NCT04621461 (Randomized; Single Group Assignment; 8 February 2021)	St. Francis Hospital (United States)
Zinc sulfate + Hydroxychloroquine in combination with doxycycline or azithromycin	Treatment (4; Completed; No results posted)	NCT04370782 (Randomized; Parallel Assignment; 30 September 2020)	St. Francis Hospital, New York (United States)
Zinc + Vitamin C + Quercetin + Bromelain	Supportive care (4; Recruiting; No results posted)	NCT04468139 (Not mentioned; Single group assignment; 30 July 2020)	Ministry of Health, Saudi Arabia (Saudi Arabia)

**Table 4:** Summary of the patent literature.

Patent/Patent Application Number (Applicant; Publication Date; Priority Country; Status *)	International Patent Classification (Family Members *)	Main Components of the Claimed Composition to Treat/Prevent COVID-19 (Ref. No.) (Use of Zinc; Clinical Studies Present/Not Present in the Example)
**WO2021216562A1** (Finzi Eric; 28 October 2021; USA; No national phase entry)	A61K31/122, A61K31/352, A61K31/353, A61K31/40, A61K31/44, A61K33/30, A61K9/20, A61P31/14 (None)	A composition of an effective amount of elemental zinc (≥100 mg/day) and a pharmaceutically acceptable adjuvant [41] (RdRp inhibitor; Present)
**US11166971B2** (Sabine Hazan; 9 November 2021; USA; Patented case)	A61K31/375, A61K31/4706, A61K31/593, A61K31/7052, A61K33/30, A61P31/14 (US2021290649A1, US2021308167A1)	A composition of hydroxychloroquine (prevents cytokine release), azithromycin (antibiotic), vitamin C, vitamin D, and zinc [42] (Vitamin C, vitamin D, and zinc provide viral protection through cellular metabolism; Present)
**US10993958B1** (William B. Coe; 4 May 2021; USA; Patented case)	A61K31/045, A61K31/11, A61K31/12, A61K31/352, A61K31/7084, A61K33/30, A61K36/48, A61K36/53, A61K36/79, A61K47/10, A61K9/00, A61K9/107,A61P31/14 (US2021283162A1)	Inhaled suspension of monoterpenoids (neral, geranial, and citronellal) as zinc ionophores, zinc, 3,3′,4′,7-tetrahydroxyflavone (metabolism booster), and 5-beta-D-ribofuranosylpicolineamide adenine-dinucleotide molecules (metabolism booster) [43] (Inhibitor of RdRp; Present)
**US10993909B1** (Virothera Pharmaceuticals; 4 May 2021; USA; Patented case)	A61K31/167, A61K31/4706, A61K31/4965, A61K31/573, A61K31/727, A61K33/30, A61K47/02, A61K47/06, A61K47/32, A61K9/00, A61K9/08, A61K9/10, A61K9/48, A61K9/50, A61K9/51 (US2021290531A1)	A composition (suspension for inhalation) comprising microparticles/nanoparticles of heparin (anticoagulant), hydroxychloroquine (anti-inflammatory agent capable of reducing the viral copy numbers of SARS-CoV-2), favipiravir (antiviral), and zinc [44] (Antiviral; Not present)
**US10987329B1** (Raju et a.; 27 April 2021; India; Patented case)	A61K31/197, A61K31/375, A61K31/5415, A61K33/30, A61K36/9066, A61K9/02 (US11026909B1)	A composition (tablet, lozenge, and suppository) of 5-aminolevulinic acid (antiviral/anticoagulant), curcumin (antioxidant), zinc, vitamin C (antioxidant and papain-like proteinase inhibitor of SARS-CoV-2), and methylene blue (antiplatelet and antiviral) [45] (Inhibitor of RNA polymerase and ACE-2; Present)
**US11026909B1** (Raju et. al.; June 8, 2021; India; Patented case)	A61K31/197, A61K31/375, A61K31/5415, A61K33/30, A61K36/9066, A61K9/02 (US10987329B1)	Claims a similar composition to US10987329B1. A pharmaceutical composition comprising 5-aminolevulinic acid, nano-curcumin, zinc, vitamin C, and methylene blue [46] (Inhibitor of RNA polymerase and ACE-2; Present)
**US11135196B1** (Matthias W. Rath; 5 October 2021; USA; Patented case)	A61K31/201, A61K31/353, A61K31/375, A61K33/30, A61K36/484, A61P31/14, A61K31/30 (WO2021206837A)	A pharmaceutical composition comprising ascorbic acid, baicalein, theaflavin, zinc aspartate, licorice, and 10-undecenoic acid [47] (All components, including zinc, lower ACE-2 expression; Not present, but cell culture studies present)
**RU2740657C1** (Promomed; 19 January 2021; Russia; Patented case)	A61K31/315, A61K31/4965, A61K31/635, A61K33/30, A61P31/14, A61P31/16 (None)	A composition (tablet, capsule, and injection) of favipiravir, darunavir, and zinc [48] (Immune booster and RNA polymerase inhibitor of SARS-CoV-2; Not present, but animal studies present)
**RU2740660C1** (Promomed; 19 January 2021; Russia; Patented case)	A61K31/4965, A61K33/30, A61P31/14, A61P31/16 (EA202190085A1)	A composition containing favipiravir and zinc [49] (Immune booster and RNA polymerase inhibitor of SARS-CoV-2; Not present, but animal studies present)
**RU2735723C1** (National Medical Research Center for Rehabilitation and Balneology; 6 November 2020; Russia; Patented case)	A61K35/60, A61K36/03, A61P31/14 (None)	Nutritional composition with nutrients including zinc [50] (Nutritional element; Not present)
**US2021402001A1** (David I. Cohen; 30 December 2021; USA; Under examination)	A61K38/17, A61K39/39, A61K47/52, A61K47/68, A61P31/14, A61P31/16, A61P35/00 (WO2022005545A1, WO2022005644A1)	A composition comprising a synergistic conjugate of a monoclonal antibody (directed at COVID-19) and a Zn-porting ionophore (alpha defensin 5) carrying a Zn cargo [51] (RNA polymerase inhibitor; Not present, but in vitro studies exemplified)
**US2021322469A1** (Regents of the University of Minnesota; 21 October 2021; USA; Under Examination)	A61K31/047, A61K31/355, A61K31/375, A61K33/30, A61K33/34, A61K9/00 (None)	An oral composition of zinc and copper (micronutrients) [52] (Inhibitor of ACE2 function; Present)
**US20210244705A1** (Centre For Digestive Diseases; 12 August 2021; USA; Under examination)	A61K31/35, A61K31/375, A61K31/47, A61K31/593, A61K31/7052, A61K33/30, A61K9/00, A61K9/14, A61K9/20 (AU2021217089A1 CA3145035A1 US2021330635A1 WO2021155443A1)	A composition (oral, injectable, or inhalation) comprising ivermectin (an antiparasitic drug with antiviral and anti-inflammatory activity), an antibiotic (doxycycline or azithromycin), and zinc [53] (Antiviral nutrient; Not present)
**US2021386779A1** (Leon Margolin; 16 December 2021; USA; Under examination)	A61K31/12, A61K31/198, A61K31/352, A61K31/355, A61K31/375, A61K31/49, A61K31/593, A61K33/30, A61K9/00, A61P31/12 (None)	A composition of zinc ionophores (quercetin, curcumin, and epigallocatechin gallate, which can enhance zinc transport into cells) and a bio-assimilable form of zinc (Zn^+2^ ion) [54] (Micronutrient/dietary supplement/RNA polymerase inhibitor; Present)
**US20210393679A1** (Melisa Institute; 23 December 2021; USA; Under examination)	A61K33/30, A61K47/54, A61P31/14 (None)	A synergistic orally effective EGCG-Zn^+2^ complex of epigallocatechin-3-gallate (EGCG) and zinc [55] (EGCG and zinc are reported as antiviral agents; Not present, but in vitro analysis is present)
**AU2020100641A4** (Balasubramaniam Vaidyanathan; 4 June 2020; Australia; Patented case)	A61K31/375, A61K31/616, A61K31/7052, A61K33/30, A61P31/12 (None)	A prophylactic regimen containing azithromycin (antibiotic), vitamin C (immune booster), zinc, and aspirin (anticoagulant) [56] (Immune booster; Not present)
**WO2021191864A1** (Dound et al.; 30 September 2021; India; No national phase entry)	A61K31/00, A61K36/00 (None)	A composition (tablet) encompassing curcumin (anti-SARS-CoV-2 compound), vitamin K2–7 (anti-SARS-CoV-2 compound), vitamin C (antioxidant), selenium, and zinc [57] (Immune-stimulant; Present)
**US2021338708A1** (Renibus Therapeutics; 4 November 2021; USA; Under examination)	A01N25/34, A01N55/02, A01N55/04, A61K31/714, A61K33/24, A61K33/30, A61K47/54, A61K9/00, A61L9/012, A61P31/14 (WO2021188787A1)	A composition of zinc protoporphyrin ix or zinc mesoporphyrin ix in combination with a pharmaceutically acceptable adjuvant [58] (Anti-COVID-19; Present)
**WO2021245365A1** (Remedy Research Limited; 9 December 2021; United Kingdom; No national phase entry)	A61K31/325, 61K31/351, A61K31/352, A61K31/4402, A61K31/4706, A61K33/04, A61K33/30, A61K45/06, A61P31/14, A61P31/16, A61P31/18 (None)	An aqueous ionic concentrate composition comprising zinc and ammonium ions (ammonium sulfate, which helps to sustain the bioavailability of zinc) [59] (RdRp inhibitor; Not present)
**WO2021234200A1** (University of Valladolid; 25 November 2021; Spain; No national phase entry)	A61K31/7048, A61K33/30, C07F3/06, C07H17/07 (ES2879148A1)	A binary complex comprising organic zinc chelate (organic zinc salt) and at least one flavonoid (silymarin, hesperidin, or diosmin) as a zinc ionophore [60] (Lowers the activity ACE-2 and RdRp enzymes; Not present)
**WO2021224836A1** (Sabharanjak Shefali; 11 November 2021; India; No national phase entry)	A61K45/06, A61K9/00 (None)	A synergistic composition comprising one zinc-chelating peptide, one protease inhibitor (9-L-arginine-peptide), and one inhibitor of viral replication enzymes (vitamin B12) [61] (Viral protein synthesis inhibitor; Not present, but in vitro assay present)
**WO2021255691A1** (Novmetapharma; 23 December 2021; USA; Under examination)	A61K31/675, A61K33/30, A61K38/05, A61K45/06, A61P31/12, A61P31/14 (US2021393731A1)	A composition of zinc and a zinc absorption enhancer, such as cyclo-His-Pro [62] (Inhibitor of replication of the RNA virus; Not present, but in vitro assays are provided)
**US2021346426A1** (Naeem Uddin and Shamail Zia; 11 November 2021; USA; Under examination)	A61K31/194, A61K33/00, A61K33/30, A61K9/00, A61K9/48, A61P31/14 (None)	A composition comprising sodium citrate (buffer), citric acid (buffer), and an effective amount of zinc [63] (Antiviral as an RNA polymerase inhibitor; Present)
**CN112402450A** (Jiangsu Techworld Medical Co. Ltd.; 26 February 2021; China; Under examination)	A61H35/04, A61K31/194, A61K33/00, A61K33/14, A61K33/30, A61K9/00, A61K9/08, A61K9/12, A61M31/00, A61P31/14, A61P31/16 (None)	A composition comprising a buffer (sodium bicarbonate, citric acid and/or malic acid, and sodium citrate and/or sodium malate), and zinc [64] (Inhibitor of the replication and reproduction of the coronavirus; Present)
**CN113208020A** (Guo Lifeng; 6 August 2021; USA; Under examination)	A23L2/38, A23L31/00, A23L33/10, A23L33/105, A23L33/15, A23L33/155, A23L33/16, A23L33/175 (None)	A plant-based health beverage comprising larch extract (dihydroquercetin), echinacea (antiviral), and *Ganoderma lucidum* (antiviral and immune booster,) along with vitamin supplements (i.e., vitamin D, vitamin E) and zinc [65] (Inhibitor of the RNA polymerase and ACE-2 of SARS-CoV-2; Not present)
**WO2021224659A1** (Aristizabal Bernal; 11 November 2021; Colombia; No national phase entry)	A61K31/085, A61K31/255, A61K31/352, A61K33/30, A61P31/14 (None)	A composition comprising allicin (antiviral), eugenol (antiviral and antioxidant), quercetin (antiviral and antioxidant), and zinc [66] (RdRp inhibitor; Not present, but in vitro antiviral activity is present)
**WO2021209652A1** (Dermopartners; 21 October 2021; Spain; No national phase entry)	A61P11/00, C07K14/79 (None)	A composition containing liposomal lactoferrin (antiviral) and zinc [67] (Immune booster and antiviral; Present)
**WO2021198908A1** (Minas Theodore Coroneo; 7 October 2021; USA; No national phase entry)	A61K31/4174, A61K31/4706, A61K31/7052, A61K31/731, A61K33/30, A61K36/04, A61K38/00, A61P31/12 (None)	A composition comprising hydroxychloroquine/chloroquine (SARS-CoV-2 inhibitor), azithromycin (antibiotic), and zinc [68] (RdRp inhibitor; Not present)
**US2022016053A1** (Zogenix International Limited; 20 January 2022; USA; Under examination)	A61K31/137, A61K31/522, A61K31/7072, A61K33/30, A61K45/06, A61P31/14 (WO2022013425A1)	A composition of a therapeutically effective amount of fenfluramine (an anorectic compound with anti-SARS-CoV-2 activity) and zinc [69] (Antiviral; Not present, but in vitro assay is exemplified)

* As of 31 January 2022.

## Data Availability

Not applicable.
